# End‐of‐life care decisions for haemodialysis patients – ‘We only tend to have that discussion with them when they start deteriorating’

**DOI:** 10.1111/hex.12454

**Published:** 2016-03-10

**Authors:** Sophia Lazenby, Adrian Edwards, Raymond Samuriwo, Stephen Riley, Mary Ann Murray, Andrew Carson‐Stevens

**Affiliations:** ^1^ Primary Care Patient Safety (PISA) Research GroupDivision of Population MedicineSchool of MedicineCardiff UniversityCardiffWalesUK; ^2^ Division of Population MedicineSchool of MedicineCardiff UniversityCardiffWalesUK; ^3^ Primary and Emergency Care Research (PRIME) Centre WalesCardiff UniversityCardiffWalesUK; ^4^ School of Healthcare SciencesCardiff UniversityCardiffWalesUK; ^5^ Cardiff Institute for Tissue Engineering and RepairCardiff UniversityCardiffWalesUK; ^6^ School of HealthcareUniversity of LeedsLeedsUK; ^7^ School of MedicineCardiff UniversityCardiffWalesUK; ^8^ Nursing Palliative Research and Education UnitFaculty of Health SciencesUniversity of OttawaOttawaONCanada; ^9^ Department of Family PracticeUniversity of British ColumbiaVancouverBCCanada; ^10^ Institute of Healthcare Policy and PracticeUniversity of the West of ScotlandPaisleyScotland

**Keywords:** advance care planning, decision making, end of life, haemodialysis, nephrologists, prognosis

## Abstract

**Background:**

Haemodialysis patients receive very little involvement in their end‐of‐life care decisions. Issues relating to death and dying are commonly avoided until late in their illness. This study aimed to explore the experiences and perceptions of doctors and nurses in nephrology for involving haemodialysis patients in end‐of‐life care decisions.

**Methods:**

A semi‐structured qualitative interview study with 15 doctors and five nurses and thematic analysis of their accounts was conducted. The setting was a large teaching hospital in Wales, UK.

**Results:**

Prognosis is not routinely discussed with patients, in part due to a difficulty in estimation and the belief that patients do not want or need this information. Advance care planning is rarely carried out, and end‐of‐life care discussions are seldom initiated prior to patient deterioration. There is variability in end‐of‐life practices amongst nephrologists; some patients are felt to be withdrawn from dialysis too late. Furthermore, the possibility and implications of withdrawal are not commonly discussed with well patients. Critical barriers hindering better end‐of‐life care involvement for these patients are outlined.

**Conclusions:**

The study provides insights into the complexity of end‐of‐life conversations and the barriers to achieving better end‐of‐life communication practices. The results identify opportunities for improving the lives and deaths of haemodialysis patients.

## Introduction

In recent years, there has been an international commitment to improve the quality and safety of health care in different contexts to ensure patients achieve the best possible health‐related outcomes.^1^, ^2^, ^3^ Quality improvement initiatives have focused on a number of domains of clinical practice to design or redesign care processes to enable patients and their families to achieve a ‘good death’.^4^, ^5^, ^6^ This has resulted in the publication of a number of guidelines in the USA^7^, ^8^ and the UK^9^, ^10^ on how to deliver end‐of‐life care for people with conditions such as end‐stage renal disease (ESRD).

Global estimates suggest the incidence and prevalence of ESRD are increasing.^11^, ^12^, ^13^, ^14^, ^15^ Annual mortality rates for ESRD are 20–25%,^16^ exceeding deaths from several cancers.^17^, ^18^ A number of studies^19^, ^20^, ^21^, ^22^ highlight there has been a significant increase in the number of people with ESRD who tend to be older (≥ 65 years), have complex health‐care needs, multiple comorbidities and are more likely to die from disease complications. Formal recommendations to enable timely palliative care for renal patients have been developed and implemented in some countries.^14^, ^23^, ^24^ There is also evidence^25^, ^26^, ^27^, ^28^, ^29^ which indicates that end‐of‐life discussions about preferences of care with haemodialysis patients are infrequently initiated by their health‐care team. One study^30^ estimates more than 50% of haemodialysis patients, who are living with ESRD, are not involved in those decisions. Emerging evidence confirms that patients want to talk about end‐of‐life and be given more information, at an earlier point in their illness,^27^, ^28^, ^29^, ^31^, ^32^ yet issues relating to death and dying are rarely considered until a medical crisis occurs.^27^, ^29^, ^30^ Some patients incorrectly believe they can be kept alive indefinitely on haemodialysis and do not view themselves as living with a terminal illness or understand that dialysis may extend life, but it might not improve their quality of life.^18^, ^28^

Involving patients in their health and treament options is recognized as a central pillar of patient‐centred care and best practice in several national and international nephrology guidelines.^23^, ^33^, ^34^, ^35^ As a growing body of evidence suggests that the quality of end of life for these patients is less than optimal, it is imperative that patients are engaged to identify their goals of care and preferences.^29^, ^36^, ^37^, ^38^ Experts in end‐of‐life care advocate earlier advance care planning, allowing patients to avoid unwanted life‐sustaining therapies and to be better prepared for death.^8^, ^25^, ^39^, ^40^, ^41^ Advance care planning is a structured and formal way of ensuring that family members and health‐care professionals are informed of the patient's preferences for subsequent care if they are unable to express a view.^22^, ^28^, ^42^, ^43^ The process of advance care planning involves identifying the patient's preferences and goals for future care through reflection and discussions with a health‐care professional that take into account the patient's values, psychological, ethical and social perspectives.^22^, ^28^, ^42^, ^43^, ^44^

Haemodialysis patients and their carers rely heavily on health‐care professionals as a source of information and support.^18^, ^24^, ^45^ With an ageing and growing dialysis population,^20^, ^21^, ^46^, ^47^ complex patients with a higher incidence of comorbidities will increasingly use nephrology services.^48^, ^49^ We explored the experiences of doctors and nurses around end‐of‐life decisions and their approaches for discussing end‐of‐life care with patients in a large nephrology and transplant unit in the UK. We report identified barriers for having end‐of‐life care discussions with patients and opportunities for improving existing communication practices in nephrology care.

## Method

Twenty semi‐structured interviews were conducted with health‐care professionals involved in the delivery of care to patients with ESRD. Individual interviews were chosen to avoid the discomfort of discussing sensitive issues in a group situation.^50^, ^51^, ^52^ A purposive stratified sample of doctors and nurses was recruited to ensure a wide representation of participants from different grades of professional standing. A snowball sample was used to gather participants through the identification of an initial subject who was used to provide the names of others. This methodology takes advantage of the social networks of identified respondents, providing an escalating set of potential contacts.^51^, ^52^, ^53^, ^54^

### Data collection

All interviews were undertaken by SL, then a medical student, using a semi‐structured topic guide that included important topics to be addressed, in all interviews, as well providing a flexible guide of open‐ended questions to explore experiences and attitudes, and allow participants to volunteer issues pertinent to them.^51^, ^52^, ^55^ The interview guide was developed through a literature review followed by construct validation with a steering group, comprising a nephrologist, a nephrology specialist nurse, and health‐care professionals and researchers from the UK and Canada with expertise in end‐of‐life decision making and shared decision making. The guide consisted of four broad topics: information giving about prognosis; discussion about advance care planning; discussion about withdrawal from dialysis; and improving end‐of‐life care. Interviews were audio‐recorded and transcribed.

### Analysis

Data were analysed using thematic analysis.^56^, ^57^ This method of analysis is essentially a process of categorizing data according to a thematic framework (or coding scheme), and key themes are summarized.^52^, ^58^ Four transcripts were independently coded by ACS and SL; subsequently, themes were compared and agreed between them and a coding framework with definitions was developed. SL subsequently applied and successively iterated the thematic framework to all data using the qualitative software package, NVivo8 (Manufactured by QSR International, Melbourne, Australia).^59^ We stopped data collection once theoretical saturation was achieved, that is, when respondents were not providing any further fresh insights.

### Ethical considerations

Ethical review was sought and granted from Cardiff University School of Medicine Research Ethics Committee (SMREC reference number: 11/48). Informed consent was obtained from all participants in this study in line with benchmarks for best practice in ethical conduct in research.^60^, ^61^, ^62^, ^63^ To obtain informed consent, all prospective participants were informed verbally and in writing about the likelihood, magnitude and duration of harm or benefit of participation in the study. They were informed that their participation in this study was voluntary and participants were free to withdraw from the study at any time. Transcripts were anonymized.

## Results

Twenty participants including seven consultants (i.e. Attendings), four specialist registrars (i.e. Fellows), four junior doctors (i.e. Residents) and five senior registered nurses were interviewed. The demographic characteristics of the participants are summarized in Table 1. Interviews lasted between 28 and 70 minutes (mean = 47 minutes).

**Table 1:** Table of demographic and professional characteris‐tics of doctors and nurses interviewed

Characteristic	Doctors	Nurses
Sex		
Female	7	5
Male	8	0
Age (years)		
Mean	37	45
Range	26–53	38–53
Years of practice in total		
Mean	13	24
Range	1.75–29	16–33
Years of practice in nephrology		
Mean	9	20
Range	0.3–26	15–26

Demographic and professional characteristics of study doctors and nurses.

Four key themes emerged from the data, including uncertainties of prognosis, the use of advance care planning in practice, limitations of dialysis withdrawal practices and barriers to achieving better end‐of‐life care (Table 2). We present the key findings for each theme.

**Table 2:** Themes and subthemes identified from interviews

Themes	Subthemes	Exemplar quote
Uncertainties of prognosis	Prognosis is not routinely given	‘They're not given a prognosis unless they actively seek it out’
	Difficulty of estimating prognosis	‘Although you know their life is going to be shorter, you can't say for that individual what their exact prognosis is going to be’
	Patients may not want or need to know about prognosis	‘I just think to give them that [prognosis] when they don't really need that. They've got enough to cope with to think they're going to be on a treatment, a long‐term treatment, a chronic treatment, until they die. And a lot of them don't want to know’
The use of advance care planning in practice	Advance care planning is rarely carried out	‘A lot of it is too little too late. You see a patient going down‐hill and then you're in a big rush to try and sort everything out’
	Initiating end‐of‐life discussions upon patient deterioration	‘It's a difficult discussion. It's easier to just let people slowly carry on and deteriorate, and they never bring it up. That's the easiest way out of it. It's a lot of effort…it's a lot harder to bring up these discussions’
Limitations of withdrawal practices	Over‐dialysis of patients	‘People say we'll try dialysis, we'll see how it goes and if they're really ill on it, we'll stop but they don't seem to stop’
	Variation in end‐of‐life care practices	‘There are some doctors who are very reluctant to withdraw and then there are others who I think are more confident and feel confident to approach a patient and say, look we should withdraw’
	Lack of talking about withdrawal before patient deterioration	‘We talk about why we're going to start it but we don't talk about why we're going to stop it, or what might be the reasons why we would want to stop it’
	Patients have a limited understanding of dialysis withdrawal	‘If you suggest that [dialysis withdrawal] and explain that they're not going to survive without dialysis, it does seem to come as quite a shock’
Barriers to achieving better end‐of‐life care	An awareness of the role of other colleagues and communication	‘Probably they ask them these questions in clinic but I don't know’
	Responsibility and culture	‘It is not knowing whose role it is and sort of passing the buck and maybe thinking, oh someone else has already spoken to them’
	Patient awareness, education and support	‘With cancer patients, you associate cancers with death…People don't associate dialysis with death as much’

### Uncertainties of prognosis

#### Prognosis is not routinely given

The length of time that a patient can expect to live following a diagnosis of ESRD is rarely discussed. Doctors perceive that they clearly signpost to patients that their life is likely to be shortened, although all doctors said specific time frames are not routinely given to patients. Whilst population‐based estimates are available, responding doctors indicated that usual practice is to give life‐expectancy information if directly asked by a patient.

They're not given a prognosis unless they actively seek it out.

(Consultant 7)

Life expectancy is usually discussed when it becomes clear a patient is declining, and in their last few weeks of life.

We only tend to have that discussion with them [about prognosis] when they start deteriorating.

(Consultant 3)

Reasons given for not opening discussions about life expectancy included the difficulty of estimating prognosis and a belief that patients did not want or need to know about prognosis.

#### Difficulty of estimating prognosis

Due to the complexity and individuality of each patient, doctors described the difficulty of trying to put a figure on the amount of time a person has left to live, admitting that it was something they felt they could not always predict.

Although you know their life is going to be shorter, you can't say for that individual what their exact prognosis is going to be.

(Consultant 2)

#### Patients may not want or need to know about prognosis

Doctors and nurses felt that patients may not want or need to know about their prognosis. It was suggested that giving prognoses may be overloading patients with too much information, and an unnecessary burden that may in fact harm patients, by causing them to become depressed.

I just think to give them that [prognosis] when they don't really need that. They've got enough to cope with to think they're going to be on a treatment – a long‐term treatment, a chronic treatment – until they die. And a lot of them don't want to know.

(Nurse 2)

However, it was noted that whilst patients may not be given a prognosis, receiving dialysis causes patients to ‘become aware of their own mortality’, having seen others die around them.

### The use of advance care planning in practice

#### Advance care planning is rarely carried out

Many participants (9/15 doctors, 5/5 nurses) expressed that advance care planning is carried out for a small proportion of patients. Some doctors described advance care planning as something they were likely to avoid, and as an issue that nobody thinks about. Reasons given for why advance care planning is rarely carried out included the difficulty of bringing up the topic of end‐of‐life, a lack of experience, a lack of policy, a focus on acute medical issues and a lack of understanding of legalities.

A lot of it is too little too late. You see a patient going down‐hill and then you're in a big rush to try and sort everything out.

(Consultant 3)

As a consequence of a lack of advance care planning, decisions are sometimes made when patients are too ill to participate.

However, others thought that advance care planning is in fact accomplished and that whilst it may be ‘ad hoc’ and ‘not very formal’, it is adequate for patient needs.

#### Initiating end‐of‐life discussions upon patient deterioration

For the majority of participants, discussions regarding end‐of‐life are initiated when an individual becomes very unwell; patient deterioration was the main trigger identified for initiating such conversations.

Despite recognizing the benefits of having earlier discussions with patients, doctors identified that in practice these are very difficult conversations to have, especially with patients who feel well, and those that are younger. As a consequence, end‐of‐life discussions are sometimes not broached until the patient has experienced significant deterioration and illness and symptom management cannot be optimized.

It's a difficult discussion. It's easier to just let people slowly carry on and deteriorate, and they never bring it up. That's the easiest way out of it. It's a lot of effort…it's a lot harder to bring up these discussions.

(Consultant 7)

### Limitations of withdrawal practices

#### Overdialysis of patients

The term ‘overdialysis’ was used by participants to describe prolonged, potentially unnecessary, treatment. Some participants (6/15 doctors and 4/5 nurses) expressed the opinion that patients can be over‐dialyzed even when they have very little quality of life and are no longer aware of their surroundings.

There are some patients who we dialyze when we perhaps shouldn't.

(Junior 2)

In particular, it was queried whether dialyzing some patients with dementia is in their best interests.

She got admitted to hospital and then the dementia rapidly worsened, she [had] many other interconnected illnesses, infections and other things. She had no concept at all, she was still given dialysis three times a week, it was very difficult because she tended to pull out the lines…

(Registrar 1)

The concept of ‘hard to stop once you've started’ was commonly used to describe the challenges in identifying goals of care and consequent actions.

People say we'll try dialysis, we'll see how it goes and if they're really ill on it, we'll stop but they don't seem to stop.

(Consultant 3)

Despite suggesting that some patients may be over‐dialyzed, doctors and nurses recognized that their perception of quality of life may be very different from that of a patient, who may want to continue dialysis for as long as possible. Furthermore, doctors emphasized that whilst it may sometimes appear patients are being over‐dialyzed, those with mild dementia can decompensate significantly during intercurrent illness and then recover a reasonable quality of life; doctors endeavour to take into account fluctuations in a patient's mental state.

#### Variation in end‐of‐life practices

Doctors reported varying approaches regarding timing of withdrawal of dialysis. Doctors and nurses disclosed that the approach to a patient's withdrawal can differ significantly depending upon the team providing care to the patient.

There are some doctors who are very reluctant to withdraw and then there are others who I think are more confident and feel confident to approach a patient and say, look we should withdraw.

(Nurse 2)

#### A lack of talking about withdrawal before patient deterioration

The majority of doctors (12/15) said they would talk about withdrawal when a patient becomes very unwell or starts to deteriorate; the possibility of withdrawal is not usually touched upon when the patient is well.

We talk about why we're going to start it but we don't talk about why we're going to stop it, or what might be the reasons why we would want to stop it.

(Consultant 4)

In my experience patients don't usually make the decision to stop dialysing. But we don't ever really discuss that with them. Usually it's just sort of presumed that everyone will keep dialysing.

(Junior 2)

#### Patients felt to have a limited understanding of dialysis withdrawal

Perhaps due to a lack of discussion about dialysis withdrawal, doctors and nurses felt that patients can have misconceptions about their possible treatment options and outcomes. Although some doctors and nurses thought that patients know they have the option to withdraw, others expressed the view that because dialysis becomes a part of a patient's everyday life they do not necessarily think that they can stop.

Once they are established on the dialysis…I don't think they are aware they can withdraw.

(Registrar 3)

It was also ascertained that some patients are not aware of the implications of stopping dialysis.

If you suggest that [dialysis withdrawal] and explain that they're not going to survive without dialysis, it does seem to come as quite a shock.

(Registrar 4)

### Barriers to achieving better end‐of‐life care

#### Awareness of the role of other colleagues and communication

Doctors and nurses were not always informed of what information is given to patients by their colleagues and consequently may not be aware of what conversations patients have had about end‐of‐life.

Probably they ask them these questions in clinic but I don't know.

(Nurse 1)

I'm not sure how many patients are told, no, I'm not aware of what the nurses actually tell them.

(Registrar 1)

Furthermore, patient preferences for end‐of‐life and registered plans of care are not consistently recorded. This was identified as a particular problem for patients admitted to hospital acutely, for whom preferences are often not known, resulting in unnecessary interventions, such as resuscitation and subsequent continuation of dialysis.

He hated being in hospital. He'd been in hospital so much he very much wanted to die at home…He had another bleed…. He was seen by a locum GP and admitted to hospital…It was not what he wanted…He was admitted to MAU, he sat on a trolley overnight, a bilateral amputee…He didn't have a registered plan of care…. The system just failed him completely.

(Consultant 5)

A lack of documentation of patient wishes was also identified as a potential cause of problems following the transition of patient care from one team to the next.

#### Responsibility and culture

There is no specified person assigned the role of having end‐of‐life discussions; thus, some individuals hope others will take responsibility for having such conversations. This is a key contributory factor towards a lack of advance care planning.

It is not knowing whose role it is and sort of passing the buck and maybe thinking, oh someone else has already spoken to them.

(Registrar 4)

Respondents indicated that raising the subject of end‐of‐life is not part of the culture in dialysis units, where it is an onerous discussion to have and not something doctors and nurses like talking about. Here, death was considered a taboo subject, and thus, it may not occur to people to discuss it. The focus instead lies on trying to keep people alive, regardless of whether or not they may have come to the end of their natural life.

It's not part of the culture…people just don't want to bring it up.

(Consultant 7)

#### Patient awareness, education and support

Participants advised patients do not associate ESRD with death in the same way patients diagnosed with cancer understand the life‐limiting implications. They suggest this may be due to a lack of discussion in the public arena and media about this issue. Doctors felt that bringing up dying with haemodialysis patients is harder, due to the patients' lack of awareness of ESRD as a life‐limiting illness.

With cancer patients, you associate cancers with death…People don't associate dialysis with death as much.

(Consultant 7)

Furthermore, whilst robust pre‐dialysis educational programmes exist, this is not always extended to patients after starting dialysis. Additionally, doctors and nurses felt that patients are not given enough personal support; in particular, there is nobody assigned the role of overseeing patients for whom dialysis is becoming less effective in managing their symptoms of advancing renal disease and may want to discuss withdrawal.

Why should we have a whole army of nurses who discuss options predialysis…why can't we have one person who is assigned that task of talking to patients who have considered withdrawal? Why can't we have the same mechanism in place for coming off dialysis?

(Consultant 5)

## Discussion

Findings from this study suggest that prognosis is not routinely discussed with patients, in part due to a difficulty in estimation of prognosis and the belief that patients do not want or need this information. There is variation of end‐of‐life practices amongst doctors. Advance care planning is carried out in moments of crisis or when a patient's condition has significantly deteriorated; thus, end‐of‐life discussions are usually initiated upon patient deterioration, and some doctors and nurses perceive patients are often withdrawn from dialysis too late. Furthermore, the possibility and implications of withdrawal are not commonly discussed with well patients.

### Relationship with the literature

Our findings are consistent with the literature,^18^, ^22^, ^26^, ^29^ in particular the issue of prognosis not being discussed in advance of patient deterioration. Previous studies have highlighted a gap in patient knowledge surrounding prognosis, particularly that they have a life‐limiting illness with a high mortality rate where only 50 per cent of dialysis patients are alive three years after starting ESRD therapy.^22^, ^64^ Doctors and nurses in our study suggest that patients did not want to be given prognoses and that to provide such information would negatively impact on patients’ well‐being. Those views are in contrast to other studies involving patient surveys and interviews, which suggest that the vast majority of patients and their carers want to be given life‐expectancy information, and for their clinician to provide this information without being prompted.^18^, ^28^, ^31^ Doctors were uncertain about the accuracy of estimating prognosis for an individual. However, developments to aid these estimations include a clinical prediction tool for six‐month mortality for patients on haemodialysis,^65^ and a clinical score to predict six‐month prognosis in elderly patients starting dialysis.^66^

There is growing evidence that advance care planning is valuable to patients with ESRD: it allows them to avoid unwanted medical interventions, help prepare themselves and those around them for death, achieve a sense of control and relieve burden placed on others.^14^, ^39^, ^42^, ^67^, ^68^, ^69^, ^70^, ^71^ However, the results of this study support previous findings that in practice patients rarely discuss end‐of‐life treatment preferences with their health‐care team, often due to the discomfort of their health‐care professionals in discussing these issues.^25^, ^26^, ^27^, ^28^, ^70^, ^72^ Doctors and nurses described a lack of experience and confidence in when to initiate and how to conduct advance care planning. Answering ‘no’ to the question, ‘Would you be surprised if this patient died within the next year?’ can be used to prompt nephrology teams to initiate end‐of‐life discussions.^73^, ^74^ This has been used extensively as a prognostic tool by providers caring for oncology, dementia and cardiovascular patients and has proven to be a good indicator of future function.^75^

Effective communication is fundamental to advance care planning which is predicated on in‐depth discussions between the health‐care professional, patient and their family to identify the patient's values, priorities and preferences for future care.^22^, ^28^, ^42^, ^44^ The participants’ accounts about a lack of discussion about end‐of‐life care amongst the health‐care team and lack of advance care planning are akin to the wider palliative care literature which refers to a similar concept colloquially called ‘conspiracy of silence’.^76^, ^77^, ^78^ In the context of palliative care, this is described as a collusion to deprive a person of information about their condition or treatment.^76^, ^77^, ^78^. For example, there is a clear gap in patient knowledge surrounding prognosis, particularly that they have a life‐limiting illness with a high mortality rate.^22^, ^64^ Evidence from a number of reviews^8^, ^79^, ^80^, ^81^, ^82^, ^83^, ^84^ has highlighted how ‘conspiracy of silence’ can result in the exclusion of patients and their families from decisions about end‐of‐life care.

A number of guidelines, communication frameworks, and tools^7^, ^8^, ^9^, ^71^, ^80^, ^85^, ^86^, ^87^, ^88^, ^89^, ^90^, ^91^ have been developed to support health‐care professionals and other members of the team such as social care professionals to develop the confidence, skills and attributes required to communicate effectively with patients and their families about end‐of‐life care. Guidance on how to conduct discussions about end‐of‐life care is provided by Davison and Torgunrud^92^, who outline a patient‐centred advance care planning model for ESRD to act as a guide to help doctors explore such issues with their patients.^92^ There are other generic approaches that can be used by health‐care teams to promote patient engagement with decisions about their end‐of‐life care; quality indicators have been outlined by Sinuff *et al*.^93^ in terms of goals for discussion, what should be documented, and organization or system issues that can aid successful planning. In addition, shared decision‐making approaches might also assist to achieve patient engagement in decision making.^33^, ^94^, ^95^

The variation in end‐of‐life decision making amongst doctors elicited from the interviews is also described in the literature.^22^, ^96^, ^97^ Differences in training have been identified as a factor underlying this variation;^98^ and it is recognized that familiarity with best practice guidelines can aid nephrology doctors in feeling more prepared for end‐of‐life decision making and reducing variability in practices such as dialysis withdrawal.^96^

### Strengths and Limitations of the study

The study provides data on an important under‐researched issue. Semi‐structured interviews are valuable for exploratory work to identify meanings and perspectives, allow the identification of cultural and social factors that influence patient care and can detect obstacles to change.^51^, ^52^, ^99^ Although interviews were undertaken with doctors and nurses, the results are intended for consideration in conjunction with previous studies focusing on eliciting patient opinions.^26^, ^27^, ^28^, ^32^, ^100^, ^101^

Data are from one large nephrology unit which limits generalizability to other settings. However, the frequency and consistency of emerging themes throughout the interviews indicate an opportunity for further work to determine whether these issues are relevant elsewhere. Whilst the study was carried out in the context of ESRD, the results may be transferable to other settings caring for patients with advanced disease.

It is recognized that it is difficult to eliminate bias and that researcher preconceptions shape research.^102^ However, the validity of the findings is supported by repeated interviewing, alongside reference to the relevant literature.

Further exploratory work is needed, including observational studies of clinician–patient interactions, and interviews with patients in the UK, to inform potential feasible interventions for improving the patient and clinician encounter around these issues. The content of current UK guidelines^23^ informing clinical decision making about haemodialysis highlights the importance of delivering patient‐centred care and enabling people in renal failure to play an active in decisions about their treatment. Approaches that use shared decision making have been shown to enable people with kidney disease to play a central role in decisions about their treatment which gave them greater confidence and control over their illness.^103^ Therefore, further research is need to examine how approaches such as shared decision making can be integrated more widely into clinical practice to bring about better patient satisfaction and improved patient outcomes.

### Recommendations for policy and practice

We have themed our recommendations broadly as better decision support for patients, better education for clinicians and better systems of care delivery. Patients and families should be supported to codesign these processes.^104^, ^105^

#### Better decision support for patients

Patients should be given realistic information about prognosis and expectations on dialysis and how they can participate in setting goals of care and be involved in care planning. Clinicians can use prognostic tools to make estimates about when to initiate discussions around end‐of‐life care.^65^, ^66^ Further, consideration should be given to provide educational and decision support for patients once they have started dialysis. Assigning a dedicated nurse to this role for patients struggling on dialysis could minimize the risk of overtreatment. Finally, public awareness of the concept of ESRD as a life‐limiting illness needs careful consideration to ensure they and their families understand the implications of their diagnosis.

#### Better education for clinicians

Doctors and nurses could benefit from support on how to initiate advance care planning discussions with their patients. The existing patient‐centred advance care planning model for ESRD could be a helpful resource for local teams to design their own systems for ensuring all patients have access to such discussions.^92^ All practising health‐care professionals should receive the appropriate preparation to achieve this pre‐ and post‐licensure, which could support acceptance of a ‘good death’ as a goal of professional practice.

#### Better systems of care delivery

Systems would need to adapt to the changes required to support the collaborative development of end‐of‐life planning between health‐care professionals and patients and their families. Registered plans of care need to become an integral part of practice and established best practice guides such as the ‘Gold Standards Framework’ can support health‐care teams in achieving this.^106^ A change in the culture of dialysis units towards ensuring all patients have access to information and an opportunity to discuss end‐of‐life decisions is needed; this will require identification of key staff to champion, lead and encourage team members to incorporate new approaches into their everyday practice.

## Conclusion

The study provides insights into the challenges of engaging in end‐of‐life conversations between patients living with ESRD and their health‐care providers. The variability in the practices of doctors and nurses is perhaps underpinned by a lack of awareness of available best evidence to inform discussions about prognosis. Whilst advance care planning is infrequently carried out, and end‐of‐life discussions are rarely initiated prior to patient deterioration, all health‐care professionals involved in this study reported the need to improve this area of practice.

## Conflict of interest

None.

## Author contributions

ACS designed the study and, together with SL, developed the coding framework. AE, SR, RS and MAM provided clinical expertise to support the analysis, particularly the contextualization of the issues identified by the research team (ACS, SL). All authors are responsible for reported research and have participated in the analysis and interpretation of data, drafting or revising, and have approved this manuscript as submitted.

## Supporting information


**Appendix S1.** Question Schedule.

## References

[1] Dixon‐Woods M , Baker R *et al* Culture and behaviour in the English National Health Service: overview of lessons from a large multimethod study. BMJ Quality & Safety, 2014; 23: 106–115. 10.1136/bmjqs-2013-001947 PMC3913222 24019507

[2] Najjar S , Hamdan M , Euwema MC *et al* The Global Trigger Tool shows that one out of seven patients suffers harm in Palestinian hospitals: challenges for launching a strategic safety plan. International Journal for Quality in Health Care, 2013; 25: 640–647. 24141012 10.1093/intqhc/mzt066

[3] Hignett S , Jones EL *et al* Human factors and ergonomics and quality improvement science: integrating approaches for safety in healthcare. BMJ Quality & Safety, 2015. In Press. 10.1136/bmjqs-2014-003623 PMC4392211 25715799

[4] Bergman B , Neuhauser D , Provost L . Five main processes in healthcare: a citizen perspective. BMJ Quality & Safety, 2011; 20 (Suppl. 1): i41–i42. 10.1136/bmjqs.2010.046409 PMC3066700 21450769

[5] Kwok AC , Semel ME , Lipsitz SR *et al* The intensity and variation of surgical care at the end of life: a retrospective cohort study. The Lancet, 2011; 378: 1408–1413. 10.1016/S0140-6736(11)61268-3 21982520

[6] Worldwide Palliative Care Alliance . Global Atlas of Palliative Care at the End of Life. London: Worldwide Palliative Care Alliance, 2014.

[7] National Consensus Project for Quality Palliative Care . Clinical Practice Guidelines for Quality Palliative Care, 3rd edn Pittsburgh, PA: National Consensus Project for Quality Palliative Care, 2013.

[8] Institute of Medicine . Dying in America Improving Quality and Honoring Individual Preferences Near the End of Life. Washington, DC: Institute of Medicine, 2014.

[9] General Medical Council . Treatment and Care Towards the End of Life: Good Practice in Decision Making. London: General Medical Council, 2010.

[10] National Institute for Health and Clinical Excellence . Quality Standard for End of Life Care for Adults. Quality staNdard Advice to the SECRETARY of State for Health. London: National Institute for Health and Clinical Excellence, 2013.

[11] Levey AS , Eckardt KU , Tsukamoto Y *et al* Definition and classification of chronic kidney disease: a position statement from Kidney Disease: Improving Global Outcomes (KDIGO). Kidney International, 2005; 67: 2089–2100. 15882252 10.1111/j.1523-1755.2005.00365.x

[12] Saran R , Li Y , Robinson B *et al* . US Renal Data System 2014 annual data report: epidemiology of kidney disease in the United States. American Journal of Kidney Diseases, 2015; 66 (1[Suppl. 1]): S1–S306. 10.1053/j.ajkd.2015.05.001 PMC6643986 26111994

[13] Murtagh FEM , Addington‐Hall J , Higginson IJ . The prevalence of symptoms in end‐stage renal disease: a systematic review. Advances in Chronic Kidney Disease, 2007; 14: 82–99. 17200048 10.1053/j.ackd.2006.10.001

[14] O'Connor NR , Corcoran AM . End‐stage renal disease: symptom management and advance care planning. American Family Physician, 2012; 85: 705–710. 22534347

[15] Smith V , Potts C , Wellard S , Penney W . Integrating renal and palliative care project: a nurse‐led initiative. Renal Society of Australasia Journal, 2015; 11: 35–40.

[16] Collins AJ , Foley RN , Herzog C *et al* Excerpts from the United States renal data system, 2008 annual data report: atlas of chronic kidney disease & end‐stage renal disease in the United States, National Institutes of Health NIDDK/DKUHD. American Journal of Kidney Diseases, 2009; 53: S1–S374. 10.1053/j.ajkd.2008.10.005 19111206

[17] Cohen LM , Moss AH , Weisbord SD , Germain MJ . Renal palliative care. Journal of Palliative Medicine, 2006; 9: 977–992. 16910813 10.1089/jpm.2006.9.977

[18] Song M‐K , Ward SE , Fine JP *et al* Advance care planning and end‐of‐life decision making in dialysis: a randomized controlled trial targeting patients and their surrogates. American Journal of Kidney Diseases, 2015; 66: 813–822. 26141307 10.1053/j.ajkd.2015.05.018 PMC5972830

[19] Murtagh FE , Marsh JE , Donohoe P , Ekbal NJ , Sheerin NS , Harris FE . Dialysis or not? A comparative survival study of patients over 75 years with chronic kidney disease stage 5. Nephrology, Dialysis, Transplantation, 2007; 22: 1955–1962. 10.1093/ndt/gfm153 17412702

[20] Van Walraven C , Manuel DG , Knoll G . Survival trends in ESRD patients compared with the general population in the United States. American Journal of Kidney Diseases, 2014; 63: 491–499. 24210591 10.1053/j.ajkd.2013.09.011

[21] Thamer M , Kaufman JS , Zhang Y , Zhang Q , Cotter DJ , Bang H . Predicting early death among elderly dialysis patients: development and validation of a risk score to assist shared decision making for dialysis initiation. American Journal of Kidney Diseases, 2015; 66: 1024–1032. 26123861 10.1053/j.ajkd.2015.05.014 PMC4658211

[22] Bristowe K , Horsley HL , Shepherd K *et al* Thinking ahead–the need for early Advance Care Planning for people on haemodialysis: a qualitative interview study. Palliative Medicine, 2015; 29: 443–450. 25527527 10.1177/0269216314560209 PMC4404401

[23] National Institute for Health and Clinical Excellence . Chronic kidney Disease Early Identification and Management of Chronic Kidney Disease in Adults in Primary and Secondary Care. London: National Institute for Health and Care Excellence, 2015. 25340245

[24] Noble H , Kelly D , Hudson P . Experiences of carers supporting dying renal patients managed without dialysis. Journal of Advanced Nursing, 2013; 69: 1829–1839. 23167619 10.1111/jan.12049

[25] Kurella TM , Cohen LM . Should there be an expanded role for palliative care in end‐stage renal disease? Current Opinion in Nephrology and Hypertension, 2010; 19: 556–560. 20644475 10.1097/MNH.0b013e32833d67bc PMC3107069

[26] Davison SN . End‐of‐life care preferences and needs: perceptions of patients with chronic kidney disease. Clinical Journal of the American Society of Nephrology, 2010; 5: 195–204. 20089488 10.2215/CJN.05960809 PMC2827591

[27] Aasen EM , Kvangarsnes M , Heggen K . Perceptions of patient participation amongst elderly patients with end‐stage renal disease in a dialysis unit. Scandinavian Journal of Caring Sciences, 2012; 26: 61–69. 21718340 10.1111/j.1471-6712.2011.00904.x

[28] Davison SN , Simpson C . Hope and advance care planning in patients with end stage renal disease: qualitative interview study. British Medical Journal, 2006; 333: 886–889. 16990294 10.1136/bmj.38965.626250.55 PMC1626305

[29] Eneanya N , Goff S , Martinez T *et al* Shared decision‐making in end‐stage renal disease: a protocol for a multi‐center study of a communication intervention to improve end‐of‐life care for dialysis patients. BMC Palliative Care, 2015; 14: 30. 26066323 10.1186/s12904-015-0027-x PMC4464129

[30] Bhargava J , Germain M , Kitsen J , Cohen LM , Meyer KB . Knowledge and participation of front‐line dialysis facility staff in end‐of‐life discussions. Nephrol News Issues, 2009; 23: 34–36, 8–40. 19753934

[31] Fine A , Fontaine B , Kraushar MM , Rich BR . Nephrologists should voluntarily divulge survival data to potential dialysis patients: a questionnaire study. Peritoneal Dialysis International, 2005; 25: 269–273. 15981775

[32] Davison SN . Facilitating advance care planning for patients with end‐stage renal disease: the patient perspective. Clinical Journal of the American Society of Nephrology, 2006; 1: 1023–1028. 17699322 10.2215/CJN.01050306

[33] Stiggelbout AM , Van der Weijden T , De Wit MPT *et al* Shared decision making: really putting patients at the centre of healthcare. BMJ, 2012; 344: e256. 22286508 10.1136/bmj.e256

[34] Coulter A , Edwards A , Elwyn G , Thomson R . Implementing shared decision making in the UK. Z Evid Fortbild Qual Gesundhwes, 2011; 105: 300–304. 21620325 10.1016/j.zefq.2011.04.014

[35] Hemodialysis Adequacy Work Group . Clinical practice guidelines for hemodialysis adequacy, update 2006. American Journal of Kidney Diseases, 2006; 48 (Suppl. 1): S2–S90. 16813990 10.1053/j.ajkd.2006.03.051

[36] Cohen LM , Germain MJ , Poppel DM , Woods AL , Pekow PS , Kjellstrand CM . Dying well after discontinuing the life‐support treatment of dialysis. Archives of Internal Medicine, 2000; 160: 2513–2518. 10979064 10.1001/archinte.160.16.2513

[37] Cohen LM , Germain M , Poppel DM , Woods A , Kjellstrand CM . Dialysis discontinuation and palliative care. American Journal of kidney Diseases, 2000; 36: 140–144. 10873883 10.1053/ajkd.2000.8286

[38] Chater S , Davison SN , Germain MJ , Cohen LM . Withdrawal from dialysis: a palliative care perspective. Clinical Nephrology, 2006; 66: 364–372. 17140166 10.5414/cnp66364

[39] Davison SN . Quality end‐of‐life care in dialysis units. Seminars in Dialysis, 2002; 15: 41–44. 11874592 10.1046/j.1525-139x.2002.00015.x

[40] Pollock K , Wilson E . Care and communication between health professionals and patients affected by severe or chronic illness in community care settings: a qualitative study of care at the end of life. Health Services and Delivery Research, 2015; 3. 26247082

[41] Barnes S , Gardiner C , Gott M *et al* Enhancing patient‐professional communication about end‐of‐life issues in life‐limiting conditions: a critical review of the literature. Journal of Pain and Symptom Management, 2012; 44: 866–879. 22819438 10.1016/j.jpainsymman.2011.11.009

[42] Luckett T , Sellars M , Tieman J *et al* Advance care planning for adults with CKD: a systematic integrative review. American Journal of Kidney Diseases, 2014; 63: 761–770. 24434187 10.1053/j.ajkd.2013.12.007

[43] Mullick A , Martin J , Sallnow L . An introduction to advance care planning in practice. BMJ, 2013; 347: f6064. 24144870 10.1136/bmj.f6064

[44] Davison SN . The ethics of end‐of‐life care for patients with ESRD. Clinical Journal of the American Society of Nephrology, 2012; 7: 2049–2057. 22997341 10.2215/CJN.03900412

[45] Gregory DM , Way CY , Hutchinson TA , Barrett BJ , Parfrey PS . Patients’ perceptions of their experiences with ESRD and hemodialysis treatment. Qualitative Health Research, 1998; 8: 764–783. 10558346 10.1177/104973239800800604

[46] Goulding MR , Rogers ME , Smith SM . Public health and aging: trends in aging – United States and worldwide (Reprinted from MMWR, vol 52, pg 101–106, 2003). Jama‐Journal American Medical Association, 2003; 289: 1371–1373.

[47] Lysaght MJ . Maintenance dialysis population dynamics: current trends and long‐term implications. Journal of the American Society of Nephrology, 2002; 13(Suppl. 1): S37–S40. 11792760

[48] Stevens LA , Viswanathan G , Weiner DE . Chronic kidney disease and end‐stage renal disease in the elderly population: current prevalence, future projections, and clinical significance. Advances in Chronic Kidney Disease, 2010; 17: 293–301. 20610356 10.1053/j.ackd.2010.03.010 PMC3160131

[49] Collins AJ , Foley RN , Chavers B *et al* United States Renal Data System 2011 Annual Data Report: Atlas of chronic kidney disease & end‐stage renal disease in the United States. American Journal of Kidney Diseases [Editorial Research Support, NIH, Extramural Research Support, US Gov't, Non‐PHS], 2012; 59 ((1 Suppl. 1):A7): e1–e420. 10.1053/j.ajkd.2011.11.015 22177944

[50] Lee RM . Doing Research on Sensitive Topics. London: Sage, 1993.

[51] Sidani S . Health Intervention Research. Understanding Research Design and Methods. London & Thousand Oaks: Sage Publications, 2015.

[52] Walker D‐M (ed). An introduction to Health Services Research. Los Angeles and London: Sage Publications, 2014.

[53] Hernon P . The sage encyclopedia of social science research methods. Library Information Scientific Research, 2004; 26: 404–406.

[54] Emmel ND . Sampling and Choosing Cases in Qualitative Research: A Realist Approach. London: Sage, 2013.

[55] Britten N . Qualitative Research .4. Qualitative Interviews in Medical‐Research. British Medical Journal, 1995; 311: 251–253. 7627048 10.1136/bmj.311.6999.251 PMC2550292

[56] Green J , Thorogood N . Qualitative Methods for Health Research. London: Sage, 2009.

[57] Braun V , Clarke V . Using thematic analysis in psychology. Qualitative Research in Psychology, 2006; 3: 77–101.

[58] Steinhauser KE , Christakis NA , Clipp EC *et al* Preparing for the end of life: preferences of patients, families, physicians, and other care providers. Journal of Pain and Symptom Management, 2001; 22: 727–737. 11532586 10.1016/s0885-3924(01)00334-7

[59] QSR International Pty Ltd . NVivo Version 8. Melbourne, Australia: QSR International Pty Ltd, 2008.

[60] Research Councils UK . RCUK Policy and Guidelines on Governance of Good Research Conduct. Swindon: Research Councils UK, 2013.

[61] Council for International Organizations of Medical Sciences (CIOMS) , the World Health Organization (WHO) . International Ethical Guidelines for Biomedical Research Involving Human Subjects. Geneva, Switzerland: Council for International Organizations of Medical Sciences (CIOMS) in collaboration with the World Health Organization (WHO), 2002.

[62] World Health Organization (WHO) . Standards and Operational Guidance for Ethics Review of Health‐Related Research with Human Participants. Geneva, Switzerland: World Health Organization (WHO), 2011. 26269877

[63] World Medical Association (WMA) . World Medical Association declaration of Helsinki: ethical principles for medical research involving human subjects. JAMA, 2013; 310: 2191–2194. 24141714 10.1001/jama.2013.281053

[64] Collins AJ , Foley RN , Chavers B *et al* ‘United States Renal Data System 2011 Annual Data Report: Atlas of chronic kidney disease & end‐stage renal disease in the United States. American Journal of Kidney Diseases, 2012; 1 (Suppl. 1): A7, e1–420. 10.1053/j.ajkd.2011.11.015 22177944

[65] Cohen LM , Ruthazer R , Moss AH , Germain MJ . Predicting six‐month mortality for patients who are on maintenance hemodialysis. Clinical Journal of the American Society of Nephrology, 2010; 5: 72–79. 19965531 10.2215/CJN.03860609 PMC2801643

[66] Couchoud C , Labeeuw M , Moranne O *et al* A clinical score to predict 6‐month prognosis in elderly patients starting dialysis for end‐stage renal disease. Nephrology, Dialysis, Transplantation, 2009; 24: 1553–1561. 10.1093/ndt/gfn698 PMC3094349 19096087

[67] Singer PA , Martin DK , Lavery JV , Thiel EC , Kelner M , Mendelssohn DC . Reconceptualizing advance care planning from the patient's perspective. Archives of Internal Medicine, 1998; 158: 879–884. 9570174 10.1001/archinte.158.8.879

[68] Martin DK , Thiel EC , Singer PA . A new model of advance care planning – Observations from people with HIV. Archives of Internal Medicine, 1999; 159: 86–92. 9892336 10.1001/archinte.159.1.86

[69] Janssen DJA , Spruit MA , Schols JMGA , van der Sande FM , Frenken LA , Wouters EFM . Insight into advance care planning for patients on dialysis. Journal of Pain and Symptom Management, 2013; 45: 104–113. 22841410 10.1016/j.jpainsymman.2012.01.010

[70] Gott M , Ingleton C , Gardiner C , Richards N , Cobb M , Ryan A *et al* Transitions to palliative care for older people in acute hospitals: a mixed‐methods study. Health Services and Delivery Research, 2013; 1. 25642530

[71] Davison SN , Kromm SK , Currie GR . Patient and health professional preferences for organ allocation and procurement, end‐of‐life care and organization of care for patients with chronic kidney disease using a discrete choice experiment. Nephrology Dialysis Transplantation, 2010; 25: 2334–2341. 10.1093/ndt/gfq072 20208074

[72] Holley JL , Hines SC , Glover JJ , Babrow AS , Badzek LA , Moss AH . Failure of advance care planning to elicit patients’ preferences for withdrawal from dialysis. American Journal of Kidney Diseases, 1999; 33: 688–693. 10196010 10.1016/s0272-6386(99)70220-9

[73] Moss AH , Ganjoo J , Sharma S *et al* Utility of the “Surprise” question to identify dialysis patients with high mortality. Clinical Journal of the American Society of Nephrology, 2008; 3: 1379–1384. 18596118 10.2215/CJN.00940208 PMC2518805

[74] Moss AH , Holley JL , Davison SN *et al* Palliative care. American Journal of Kidney Diseases, 2004; 43: 172–185. 14712442 10.1053/j.ajkd.2003.12.009

[75] Lynn J , Schall MW , Milne C , Nolan KM , Kabcenell A . Quality improvements in end of life care: insights from two collaboratives. Joint Commission Journal on Quality Improvement, 2000; 26: 254–267. 18350770 10.1016/s1070-3241(00)26020-3

[76] Chaturvedi SK , Loiselle CG , Chandra PS . Communication with relatives and collusion in palliative care: a cross‐cultural perspective. Indian Journal of Palliative Care, 2009; 15: 2–9. 20606848 10.4103/0973-1075.53485 PMC2886207

[77] Fallowfield LJ , Jenkins VA , Beveridge HA . Truth may hurt but deceit hurts more: communication in palliative care. Palliative Medicine, 2002; 16: 297–303. 12132542 10.1191/0269216302pm575oa

[78] Neuberger J , Guthrie C , Aaronovitch D *et al* More Care, Less Pathway. A Review of the Liverpool Care Pathway. London: HMSO, 2013.

[79] Department of Health . Government Response to the House of Commons Health Select Committee Report on End of Life Care. (Fifth report of session 2014–15) London: Department of Health, 2015.

[80] The Choice in End of Life Care Programme Board (ELCPB) . What's Important to Me. A Review of Choice in End of Life Care. Leeds: The Choice in End of Life Care Programme Board, 2015.

[81] Giovanni LA . End‐of‐life care in United States: current reality and future promise–a policy review. Nursing Economics, 2012; 30: 127–134 quiz 35. 22849010

[82] The House of Commons Public Administration and Constitutional Affairs Committee (PACC) . Follow‐up to PHSO Report: Dying without dignity. London: The Stationery Office Limited on behalf of the House of Commons Public Administration and Constitutional Affairs Committee, 2015.

[83] Parliamentary and Health Service Ombudsman (PHSO) . Dying Without Dignity. Investigations by theParliamentary and Health Service Ombudsman into Complaints about End of Life Care. London: Parliamentary and Health Service Ombudsman, 2015.

[84] Schüklenk U , Van Delden JJM , Downie JM , McLean SAM , Upshur R , Weinstock D . End‐of‐life decision‐making in Canada: the report by the royal society of Canada expert panel on end‐of‐life decision‐making. Bioethics, 2011; 25: 1–73. 10.1111/j.1467-8519.2011.01939.x PMC3265521 22085416

[85] National Institute for Health and Care Excellence (NICE) . End of Life Care for Adults. London: National Institute for Health and Care Excellence, 2011.

[86] National Council for Palliative Care (NCPC) . The end of Life Care Strategy: New Ambitions. London: National Council for Palliative Care, 2013.

[87] National Council for Palliative Care (NCPC) . A National Review of Choice in End of Life Care – Consultation Paper. London: National Council for Palliative Care, 2014.

[88] NHS England . Developing a New Approach to Palliative Care Funding: A First Draft for Discussion. Leeds: Palliative Care Team, 2014.

[89] NHS England . Actions for End of Life Care: 2014–16. London: NHS England, 2014.

[90] McCutcheon AK , Kabcenell A , Little K , Sokol‐Hessner L . “Conversation Ready”: A Framework for Improving End‐of‐life Care. Cambridge, Massachusetts: Institute for Healthcare Improvement; 2015. (Available at ihi.org), 2015.

[91] Scottish Partnership for Palliative Care (SCPC) . Living and Dying Well. Edinburgh: Scottish Partnership for Palliative Care, 2015 Available at: http://www.palliativecarescotland.org.uk/content/living_dying_well/, accessed 4 December 2015.

[92] Davison SN , Torgunrud C . The creation of an advance care planning process for patients with ESRD. American Journal of Kidney Diseases, 2007; 49: 27–36. 17185143 10.1053/j.ajkd.2006.09.016

[93] Sinuff T , Dodek P , You JJ *et al* Improving end‐of‐life communication and decision making: the development of a conceptual framework and quality indicators. Journal of Pain & Symptom Management, 2015; 49: 1070–1080. 25623923 10.1016/j.jpainsymman.2014.12.007

[94] Joseph‐Williams N , Elwyn G , Edwards A . Knowledge is not power for patients: a systematic review and thematic synthesis of patient‐reported barriers and facilitators to shared decision making. Patient Education and Counseling, 2014; 94: 291–309. 24305642 10.1016/j.pec.2013.10.031

[95] Elwyn G , Frosch D , Thomson R *et al* Shared decision making: a model for clinical practice. Journal of General Internal Medicine, 2012; 27: 1361–1367. 22618581 10.1007/s11606-012-2077-6 PMC3445676

[96] Holley JL , Davison SN , Moss AH . Nephrologists’ changing practices in reported end‐of‐life decision‐making. Clinical Journal of the American Society of Nephrology, 2007; 2: 107–111. 17699394 10.2215/CJN.03080906

[97] McKenzie JK , Moss AH , Feest TG , Stocking CB , Siegler M . Dialysis decision making in Canada, the United Kingdom, and the United States. American Journal of Kidney Diseases, 1998; 31: 12–18. 9428446 10.1053/ajkd.1998.v31.pm9428446

[98] Davison SN , Jhangri GS , Holley JL , Moss AH . Nephrologists’ reported preparedness for end‐of‐life decision‐making. Clinical Journal of the American Society of Nephrology, 2006; 1: 1256–1262. 17699356 10.2215/CJN.02040606

[99] Pope C , Ziebland S , Mays N . Analysing qualitative data In: Pope C, Mays N (eds) Qualitative Research in Health Care, 2nd edn London: BMJ Books, 2000. 10.1136/bmj.320.7227.114 PMC1117368 10625273

[100] Axelsson L , Randers I , Jacobson SH , Klang B . Living with haemodialysis when nearing end of life. Scandinavian Journal of Caring Sciences, 2012; 26: 45–52. 21605154 10.1111/j.1471-6712.2011.00902.x

[101] Elliott BA , Gessert CE , Larson P , Russ TE . Religious beliefs and practices in end‐stage renal disease: implications for clinicians. Journal of Pain and Symptom Management, 2012; 44: 400–409. 22762965 10.1016/j.jpainsymman.2011.09.019

[102] Cohen DJ , Crabtree BF . Evaluative criteria for qualitative research in health care: controversies and recommendations. Annals of Family Medicine, 2008; 6: 331–339. 18626033 10.1370/afm.818 PMC2478498

[103] The Health Foundation . Person‐centred Care Resource Centre. Spotlight on Renal Care. London: The Health Foundation 2015 [cited 2015 13 December]. Available from: http://personcentredcare.health.org.uk/renal.

[104] Locock L , Robert G , Boaz A , Vougioukalou S , Shuldham C , Fielden J *et al* Testing accelerated experience‐based co‐design: a qualitative study of using a national archive of patient experience narrative interviews to promote rapid patient‐centred service improvement. Health Services and Delivery Research, 2014; 2. 25642558

[105] Robert G , Cornwell J , Locock L , Purushotham A , Sturmey G , Gager M . Patients and staff as codesigners of healthcare services. BMJ, 2015; 350: g7714. 25670179 10.1136/bmj.g7714

[106] Thomas K . The Gold Standards Framework: A Programme for Community Palliative Care. Shrewsbury: NHS End of Life Care Programme, 2005.

