# Supplementary material for: End‐of‐life care decisions for haemodialysis patients – ‘We only tend to have that discussion with them when they start deteriorating’
Source: Health Expect. 2016 Mar 10;20(2):260–73. doi: 10.1111/hex.12454 (PMC5354044; doi:10.1111/hex.12454)
Supplement: Supplementary file 1 — Appendix S1. Question Schedule. [file HEX-20-260-s001.docx]

**Appendix 1: Question Schedule**

1. **Reflection upon a patient situation.**

Have you been in/witnessed any situation/scenario involving a patient on haemodialysis nearing the end of their life which particularly sticks in your mind? What was good/bad? What would you have done differently?

1. **Experiences and approaches used in giving information about prognosis.**

| Questions: prognosis. |
| --- |
| 1. What information are patients given about their renal disease prognosis and renal disease process? |
| 2. Do you think patients are given the appropriate amount of information? (Prompt: are patients given too much, the right amount or not enough information about prognosis and the disease process?) |
| 3. At what point in a patient’s care do you usually have discussions with the patient about prognosis and the future? |
| 4. When do you think would be the optimal time for these discussions to occur? (Prompt: do you think it would be more beneficial for the patient for these discussions to occur earlier or later? Do you think patients should be informed about their prognosis before, when starting, during or towards the end of dialysis?) |
| 5. Should patients be given life expectancy information? Why do you think that? |
| 6. How likely are you to give a patient life expectancy information without being asked specifically for this information? |
| 7. Tell me some of the reasons that might persuade you to give life expectancy information |
| 8. Tell me some of the reasons why you might not give the patient life expectancy information |

1. **Experiences and approaches regarding advance care planning.**

| Questions: advance care planning. |
| --- |
| 1. Who should initiate discussions relating to end-of-life issues? (Prompt: do you believe it is better for you or the patient to initiate discussions relating to end-of-life issues?) |
| 2. Do you ever feel reluctant to discuss end-of-life issues, or find it difficult (related to end-stage kidney disease)? |
| 3. What kind of things would prompt you to talk about end-of-life with a patient? |
| 4. What factors make you less likely to talk about advance care planning with a patient/ what makes it difficult for you to talk about end-of-life issues? (Prompt: do you ever feel that advance care planning/end-of-life discussions take away a patient’s hope?) |
| 5. Do you ever discuss the role that the patient and their family would like to have with regards to advance care planning and making end-of-life decisions? If so, in what proportion of patients do you do this? |
| 6. In what proportion of patients do you feel advance care planning is adequately carried out? |
| 7. What do you feel are the main barriers to achieving better advance care planning? |

1. **Experiences and approaches with regards to withdrawal from dialysis.**

| Questions: Withdrawal from dialysis |
| --- |
| Do you think that most patients are aware that they have the option to discontinue from dialysis at any point? If no, what might be the reasons for this? |
| How much involvement do family members have in a relative’s withdrawal from dialysis? |
| What concerns do you think family members have about their relative’s withdrawal from dialysis and end-of-life? |
| At what point in patient’s care do you think the option to withdraw from dialysis should be discussed? |
| In what situations would you talk about withdrawing from dialysis with a patient? What factors or situations might cause you to delay talking about withdrawal? |

1. **Improving end-of-life care.**

| Questions: improving end-of-life care. |
| --- |
| 1. Can we improve the way in which we help ESRD patients to make end-of-life decisions? If yes, how can this be improved? If no, why can it not be improved? |
| 2. What information do you think the patient should be provided with in end-of-life discussions? Follow up: Of the things that you said, what are the most essential components that should be included in an information/support intervention to help patients make end-of-life decisions? |
| 3. Imagine you are going to see an ESRD patient now – what kind of questions would you ask them to elicit their goals, preferences, expectations, fears and desires about the future? |
